# Transient, small‐scale field‐aligned currents in the plasma sheet boundary layer during storm time substorms

**DOI:** 10.1002/2016GL068768

**Published:** 2016-05-30

**Authors:** R. Nakamura, V. A. Sergeev, W. Baumjohann, F. Plaschke, W. Magnes, D. Fischer, A. Varsani, D. Schmid, T. K. M. Nakamura, C. T. Russell, R. J. Strangeway, H. K. Leinweber, G. Le, K. R. Bromund, C. J. Pollock, B. L. Giles, J. C. Dorelli, D. J. Gershman, W. Paterson, L. A. Avanov, S. A. Fuselier, K. Genestreti, J. L. Burch, R. B. Torbert, M. Chutter, M. R. Argall, B. J. Anderson, P.‐A. Lindqvist, G. T. Marklund, Y. V. Khotyaintsev, B. H. Mauk, I. J. Cohen, D. N. Baker, A. N. Jaynes, R. E. Ergun, H. J. Singer, J. A Slavin, E. L. Kepko, T. E. Moore, B. Lavraud, V. Coffey, Y. Saito

**Affiliations:** ^1^ Space Research InstituteAustrian Academy of SciencesGrazAustria; ^2^ Earth Physics DepartmentSt. Petersburg State UniversitySt. PetersburgRussia; ^3^ Institute of Geophysics and Planetary Physics and Earth and Space Sciences DepartmentUniversity of CaliforniaLos AngelesCaliforniaUSA; ^4^ Goddard Space Flight CenterNASACollege ParkMarylandUSA; ^5^ Denali ScientificHealyAlaskaUSA; ^6^ Southwest Research InstituteSan AntonioTexasUSA; ^7^ University of Texas at San AntonioSan AntonioTXUSA; ^8^ Physics DepartmentUniversity of New HampshireDurhamNew HampshireUSA; ^9^ Applied Physics LaboratoryThe Johns Hopkins UniversityLaurelMarylandUSA; ^10^ Space and Plasma PhysicsRoyal Institute of TechnologyStockholmSweden; ^11^ Swedish Institute of Space PhysicsUppsalaSweden; ^12^ University of ColoradoBoulderColoradoUSA; ^13^ NOAA Space Weather Prediction CenterBoulderColoradoUSA; ^14^ Department of Climate and Space Sciences and EngineeringUniversity of MichiganAnn ArborMichiganUSA; ^15^ Centre National de la Recherche ScientifiqueToulouseFrance; ^16^ NASA Marshall Space Flight CenterHuntsvilleAlabamaUSA; ^17^ JAXA Institute for Space and Astronautical ScienceSagamiharaJapan

**Keywords:** field‐aligned currents, electron beam, MMS, PSBL

## Abstract

We report on field‐aligned current observations by the four Magnetospheric Multiscale (MMS) spacecraft near the plasma sheet boundary layer (PSBL) during two major substorms on 23 June 2015. Small‐scale field‐aligned currents were found embedded in fluctuating PSBL flux tubes near the separatrix region. We resolve, for the first time, short‐lived earthward (downward) intense field‐aligned current sheets with thicknesses of a few tens of kilometers, which are well below the ion scale, on flux tubes moving equatorward/earthward during outward plasma sheet expansion. They coincide with upward field‐aligned electron beams with energies of a few hundred eV. These electrons are most likely due to acceleration associated with a reconnection jet or high‐energy ion beam‐produced disturbances. The observations highlight coupling of multiscale processes in PSBL as a consequence of magnetotail reconnection.

## Key Points

- Multipoint multiscale observations of field‐aligned currents during storm time substorms
- Small‐scale intense field‐aligned currents are found embedded in PSBL flux tubes near separatrix region
- Field‐aligned low‐energy electron beams correlate with short‐lived localized field‐aligned currents

## 1 Introduction

In the Earth's magnetotail the most dynamic energy conversion takes place during substorms. Reconfiguration of the magnetotail current sheet and enhanced coupling to the ionosphere occur, driven by near‐Earth magnetotail reconnection and other current sheet instabilities. Key features of substorms are plasma sheet thinning and expansion, magnetic field dipolarization, enhanced occurrence of bursty bulk flows, energetic particle injection, and intensified field‐aligned currents (FACs) including the formation of the substorm current wedge. Recent advances in our understanding of substorms based on multipoint spacecraft observations and simulations are discussed by *Sergeev et al.* [2012].

The plasma sheet boundary layer (PSBL), between the hot plasma sheet and the tenous lobe, is a dynamic region in the magnetotail where counterstreaming ions are often observed independent of geomagnetic activity [*Eastman et al.*, 1984]. During substorm onset, a spacecraft located near the PSBL usually experiences a sudden dropout of hot plasma sheet plasma, entering the lobe due to thinning of the plasma sheet. *Hones et al.* [1984] considered that this fast thinning at substorm onset was due to the formation of a reconnection region earthward of the spacecraft, whereas the subsequent plasma sheet expansion was due to its tailward retreat. *Sergeev et al.* [2008], however, reported that both the thinning of the plasma sheet and dipolarization can take place earthward of a near‐Earth closed‐field line reconnection region. The subsequent expansion of the plasma sheet was interpreted to be due to the enhanced reconnection rate as the reconnection started to involve lobe field lines, causing rapid earthward transport of the heated plasma sheet plasma. At shorter time scales, plasma sheet thinning and thickening were also observed during bouncing of earthward flows [e.g., *Panov et al.*, 2010]. One way to distinguish these different scenario is to examine the ion flows at the plasma sheet exit/entry. The near‐Earth PSBL disturbances during substorms therefore reflect the evolution of the near‐Earth reconnection processes.

In this paper we report on the evolution of the FACs during a storm time substorm interval on 23 June 2015, when the four Magnetospheric Multiscale (MMS) [*Burch et al.*, 2015] satellites were located near the southern edge of the near‐Earth plasma sheet. Using the high time resolution measurements from MMS, we resolve, for the first time, intriguingly detailed properties of small‐scale FACs in the PSBL.

## 2 Storm Time Substorms

**Figure 1:**
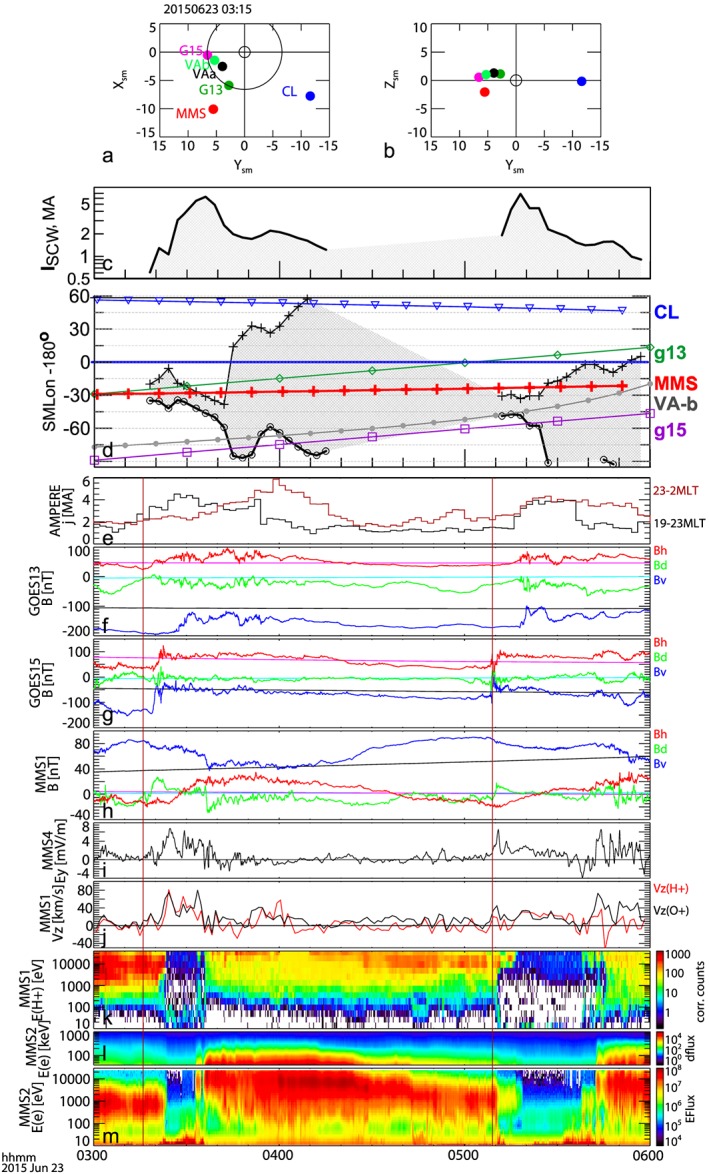
Overview of two substorms on 23 June 2015. Spacecraft location in the (a) *X*‐*Y* and (b) *Y*‐*Z* plane in the solar magnetic (SM) coordinate system. (c) Total current of the substorm current wedge (SCW) and (d) local time of the SCW and different spacecraft. (e) Premidnight (black) and postmidnight (red) field‐aligned current obtained from AMPERE. Magnetic fields in *V* *D* *H* coordinate system from (f) GOES13, (g) GOES15, and (h) MMS1. The observed *B*_*V*_, *B*_*D*_, and *B*_*H*_ components are plotted in blue, green, and red, whereas the three components predicted from the T89 model are plotted in black, cyan, and pink, respectively. (i) Dawn‐to‐dusk component of the spin‐averaged electric fields from MMS4 and (j) northward flow velocity from proton (red) oxygen (black) from MMS1 in near‐geocentric solar ecliptic (GSE) coordinate system. Differential energy flux from (k) protons, (l) energetic electrons, and (m) electrons from MMS2. The vertical lines indicate the 03:16 UT and 05:09 UT onsets.

On 23 June 2015, two major substorm expansion phase onsets commenced at 03:16 and 05:09 UT. These occurred when MMS satellites were traversing the premidnight magnetosphere under a fortuitous conjunction with a number of spacecraft in the nightside magnetosphere: Geostationary Operational Environmental Satellites (GOES), MMS, Cluster, Van Allen Probes, and Active Magnetosphere and Planetary Electrodynamics Response Experiment (AMPERE), as shown in Figures 1a and 1b. The substorms took place during a strong geomagnetic storm, which followed the arrival of an interplanetary shock at 18:36 UT on 22 June 2015 (e.g., Reiff et al., private communications, 2016). The *D* *s* *t* index reached −204 nT at 4 UT on 23 June. For both substorms, multiple westward electrojet intensifications were detected in high‐latitude ground‐based magnetograms (Figure S1 in the supporting information). Enhancements of field‐aligned currents (FACs) with intensities reaching 6–7 MA occurred following these onsets. The enhancements were centered in the premidnight local time sector as inferred from a substorm current wedge model [*Sergeev et al.*, 2011] using midlatitude magnetograms (Figures 1c and d) and also the integrated FAC obtained from the AMPERE [*Anderson et al.*, 2000] dat (Figure 1e).

Figures 1f and 1g show the magnetic field disturbances observed by GOES 13 and 15 [*Singer et al.*, 1996] plotted in *V* *D* *H* coordinate, where *H* is parallel to the geomagnetic dipole axis and positive northward. *D* is in the direction of *H* × *R* and is positive eastward, where *R* is the radius vector from the center of the Earth to the spacecraft. *V* is parallel to the magnetic equatorial plane and radially outward from the Earth. Both substorm onsets were accompanied by dipolarization (enhancement in the *B*_*H*_ component) at GOES, starting from a highly stretched tail‐like field configuration, as can be seen by the nearly 100 nT deviation of the *B*_*V*_ component from the T89 model for Kp5 [*Tsyganenko*, 1989]). Both substorms have (1) multiple dipolarizations visible at GOES, (2) multiple enhancements of the westward electrojet (Figure S1), and (3) widening of the extent of the current wedge in local time as compared to the initial premidnight location (Figure 1d).

MMS was located at a local time near the center of the current wedge for both onsets (Figure 1d). Magnetic field data from the MMS fluxgate magnetometer [*Russell et al.*, 2014] are shown in Figure 1h. The large positive *B*_*V*_ component, as expected from the MMS location in the Southern Hemisphere, started to decrease in association with the 03:16 onset as the tail‐like configuration changed to a dipolar configuration, also identified from enhancement in the *B*_*H*_ component. The observed sharp *B*_*D*_ disturbances suggest enhancements in the FACs. In spite of a more dipolar configuration after the 03:16 UT onset, detected by GOES and MMS in both hemispheres, the MMS spacecraft entered the lobe at around 03:23 UT. Evidence of the lobe plasma includes the energy spectra of protons detected by the Hot Plasma Composition Analyzer (HPCA) [*Young et al.*, 2014] (Figure 1k) and electron data from the Energetic Ion Spectrometer (EIS) [*Mauk et al.*, 2014] (Figure 1l) and Fast Plasma Instruments (FPIs) [*Pollock*, 2016] (Figure 1m). Such a dropout of the plasma sheet population may suggest a reconfiguration of the current sheet structure by near‐Earth reconnection in the closed‐field region [e.g., *Sergeev et al.*, 2008]. Consistently, ion flow moment during PSBL exit had earthward component (not shown). The enhanced dawn‐to‐dusk electric field (Figure 1i) from the MMS Spin‐Plane Double Probe electric field instrument (SDP) [*Lindqvist et al.*, 2014] and the northward flows are evidence for enhanced inward motion of cold oxygen and protons in the lobes (Figure 1j) toward the plasma sheet. This evidence suggests an enhanced reconnection rate supporting the above interpretation. The cold ions are detected as a narrow energy band enhancement in the energy spectra of H^+^ (Figure 1k) at a level comparable to the drift energy as was reported in previous observations [*Sauvaud et al.*, 2004]. A velocity of 70 km/s, for example, would correspond to a proton energy of 24 eV, which is comparable to the observed level of the cold protons visible in Figure 1k and velocity in Figure 1j and the electric field (4.2 mV/m for 60 nT) in Figure 1i. Although not shown, substantial cold O^+^ was also present.

Around 03:35 UT, associated with another westward electrojet enhancement (Figure S1) MMS reentered the plasma sheet accompanied by a high‐energy particle injection (Figure 1l), a sharp change in the *B*_*V*_ and *B*_*D*_, and enhanced disturbances in the *B*_*H*_ (Figure 1h), when GOES also observed another enhancement in *B*_*H*_ (Figures 1f and 1g). Reentry into the plasma sheet is therefore due to another reconfiguration of the current sheet, i.e., expansion of the plasma sheet with more dipolar configuration, observed both in the Northern and Southern Hemispheres. The second substorm onset at 05:09 UT showed similar multiple activations of the westward electrojet, dipolarization at GOES 15 in the premidnight region, and a dipolarization at MMS with plasma sheet thinning and expansion during the first and second onset, respectively. For both events the PSBL that reentered from the lobe is highly energized and accompanied by a more disturbed magnetic field compared to the PSBL preceding the lobe interval.

## 3 Plasma Sheet Boundary Layer Crossings

**Figure 2:**
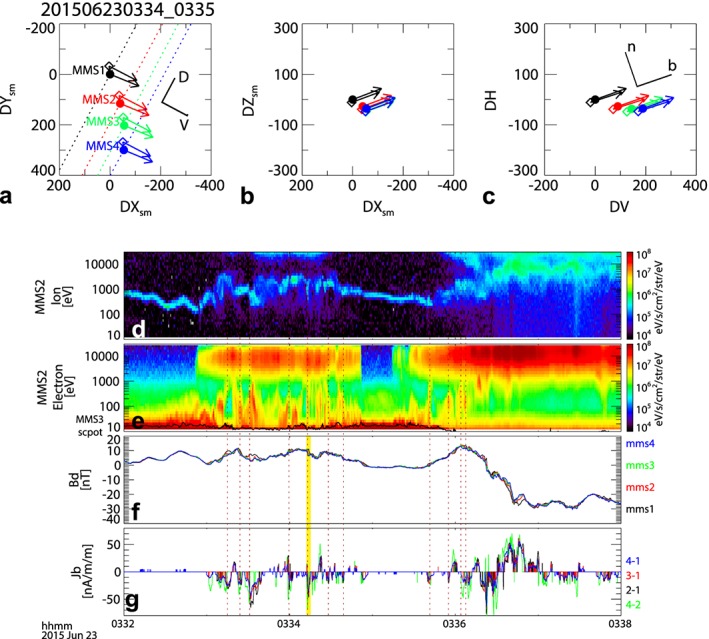
MMS four spacecraft configuration and the plasma and magnetic field observations during plasma sheet expansion between 03:32 and 03:38 UT. Location of the four MMS spacecraft (relative to MMS1 location at 03:34 UT) in (a) *X*‐*Y* and (b) *X*‐*Z* plane in the SM coordinate system and in the (c) *H* − *V* plane for 03:34 UT (solid circles) and 03:35 UT (diamonds). The 1 min averaged magnetic field vectors are indicated as arrows. The differential energy flux of (d) ions and (e) electrons from MMS2 and (f) *B*_*D*_ component of the magnetic field from the four MMS spacecraft and (g) current density along the average magnetic field direction, *J*_*b*_, estimated from pairs of MMS spacecraft given in the labels. Derivation of the current density is explained in the text. The black curve in Figure 2e shows the spacecraft potential from MMS3. The red dotted lines indicate the small‐scale downward FAC events accompanied by the low‐energy electron signatures. The yellow vertical line indicates the 03:34:12 UT event.

Figure 2 shows the changes in the plasma and the magnetic fields between 03:32 and 03:38 UT during the reentry of the plasma sheet. The relative location of the four spacecraft and 1 min average magnetic field vectors for 03:34 and 03:35 UT are depicted in Figures 2a–2c. The four MMS spacecraft were on an inbound orbit, traversing mainly in dawnward direction and forming a string‐of‐pearls configuration led by MMS1, followed by MMS2, MMS3, and MMS4. The largest spacecraft separation was about 300 km, mainly along the dawn‐dusk direction in the solar magnetic coordinate (SM) system (Figures 2a and 2b). The average magnetic field direction is in the *V* − *H* plane and is tilted from the *H* axis about 70°, i.e., predominantly tailward, as expected in the PSBL in the Southern Hemisphere. We can therefore conclude that the background magnetic field configuration can be well organized with the *V* *D* *H* coordinate system. MMS1 is then located at the most earthward (equatorward) flux tube, while MMS4 is at the most tailward (outward) one.

Figures 2d and 2e show the 0.5 s averaged ion and electron omnispectra from the FPI instrument. The solid curve in Figure 2e shows the spacecraft potential from MMS3. The entry into the plasma sheet first took place around 03:33 UT and after a short exit again at around 03:35:30 UT. The entry is clearly visible in the electron spectra, while the energy of the ions in the plasma sheet exceeds the FPI instrument threshold, and hence, only the low‐energy part of the high‐energy ion population is seen. The narrow energy band beams seen in the range between 100 and 1000 eV are actually from oxygen, identified by the HPCA instrument (not shown). The appearance of the plasma sheet population is directly related to the enhancement of the energy of the cold population and, hence, enhancements in the drift energy and in the electric field.

Figure 2f shows the *B*_*D*_ component of the magnetic field, the most variable component during the time interval of the second plasma sheet entry, in Figure S2. The angle between maximum variance and *D* direction is within 25° for both thinning and expanding plasma sheet intervals (Table S1). Note that the magnetic field components compared between different spacecraft (Figure S2, bottom three panels) differ most or show the largest variations also for the *B*_*D*_ component. This indicates that *B*_*D*_ is the overall maximum varying component both in space and time. Hence, the magnetic field variations can be treated as a planar current sheet and we can estimate the current density using the *B*_*D*_ differences obtained by the pairs of spacecraft. This 2SC method is particularly useful for this event since a string‐of‐pearls configuration of the four spacecraft does not allow the conventional 3‐D linear gradient analysis techniques to determine the gradient of the field.

We estimated the FAC density, *J*_*B*_, from the gradient of *B*_*D*_ along the *N* direction determined from the pairs of spacecraft, Δ*B*_*D*_/Δ*r*_*N*_. Here we consider a local FAC coordinate system, *B* *D* *N*. *N* is directed to *D* × *B*_0_, where *B*_0_ is the background magnetic field. For the background magnetic field we used 1 min averaged values between 03:34 UT and 03:35 UT. *B* closes the rectangular coordinate system. The vectors defining this FAC coordinate system are given in Table S2. Since ***B*_0_** and *B* deviate by less than 5.3°, *B* is essentially the direction parallel to the background magnetic field. Taking into account the limitation in calibration of the commissioning phase magnetic field data in terms of spin‐axis offset, we estimate the current density only when the difference in the magnetic field component between two spacecraft exceeded 0.5 nT. Figure 2g shows that at the outer edge of the PSBL, the currents are highly structured and the peaks are mainly toward the Earth (negative *J*_*B*_). Many of those downward currents are also well correlated with the spiky enhancements in the low‐energy electrons (Figure 2e) as indicated by the dotted vertical lines. Between 03:36 and 03:37 UT, earthward (or downward) currents change to tailward (or upward) currents as the spacecraft reentered the plasma sheet. A reversal of the current associated with the crossing of the plasma sheet boundary layer in the expanding plasma sheet during active times is consistent with previous observations [*Nakamura et al.*, 2004; *Grigorenko et al.*, 2009] of crossing of the separatrix layer of an active X line. What these previous observations could not resolve, however, are the highly structured FACs and the corresponding electron signatures due to the limitation in temporal and spatial resolution.

## 4 Transient/Localized Field‐Aligned Currents

**Figure 3:**
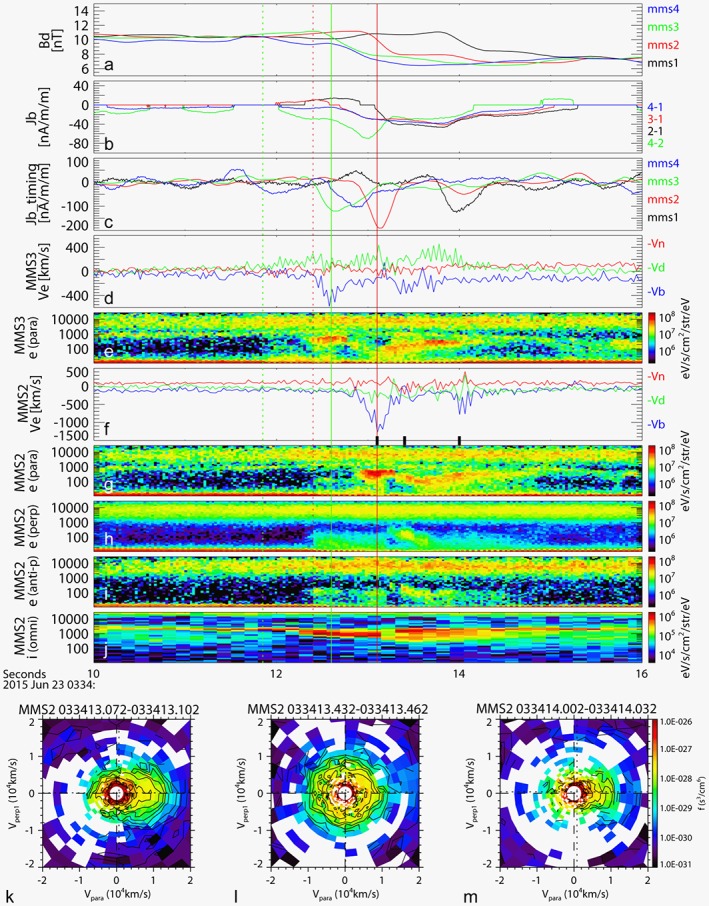
Magnetic field and plasma observations between 03:34:10 and 03:34:16 UT. (a) *B*_*D*_ component from the four MMS spacecraft. (b) FAC, *J*_*b*_, determined from pairs of MMS spacecraft. (c) FAC, *J*_*b*_, determined from single spacecraft using timing velocity. Electron velocity moment in *b* *d* *n* coordinate system from (d) MMS3 and (f) MMS2. Pitch angle sorted electron energy spectra for pitch angle between 0° and 20° for (e) MMS3 and (g) MMS2, (h) between 70° and 110° and (i) between 160° and 180° for MMS2 and (j) ion omnidirectional energy spectra from MMS2. Two‐dimensional cut of the velocity distribution function for MMS2 in the plane of the magnetic field *V*_par_ and the perpendicular flow direction *V*_perp1_, which is perpendicular to the *V* × *B* direction for three selected time intervals (k) 03:34:13.072, (l) 03:34:13.432, and (m) 03:34:14.002 as indicated as black bars in Figure 3f. The vertical solid lines show the time of the current density peak for MMS3 (green) and MMS2 (red), while the dashed lines show the start of the very low energy electron enhancements.

Figure 3 shows the magnetic field and particle data between 03:34:10 UT and 03:34:16 UT during one of the short earthward current interval starting from around 03:34:12 UT (marked by a yellow line in Figure 2). The *B*_*D*_ component of the four spacecraft (Figure 3a) shows a two‐step decrease. These similar profiles among the four spacecraft are expected for a planar geometry. The profiles of MMS3, MMS2, and MMS1 are almost identical. MMS4 observed also two‐step changes in *B*_*D*_ preceding the other three spacecraft, but unlike the other three spacecraft, the second *B*_*D*_ change is more pronounced than the first one. We estimate the propagation speed from the timing of the peak in the *B*_*D*_ gradient from MMS1, MMS2, and MMS3 to be 62 km/s along the positive *N*, directed equatorward‐earthward. This timing velocity corresponds to velocity of O^+^ with kinetic energy of 300 eV, which is comparable to the energy flux enhancement visible in Figure 3j. It is also comparable to the average northward ion velocity shown in Figure 1j (and the corresponding dawn‐to‐dusk electric field of about 4 mV/m in Figure 1i). Hence, the four MMS spacecraft are monitoring a FAC sheet convecting equatorward with an enhanced dawn‐to‐dusk electric field. If we assume a planar FAC sheet, the observed time scale of the *B*_*D*_ changes (about 0.4 s) correspond to a FAC sheet with a thickness of about 25 km. The fact that the scale size of the current sheet is well below (2–5 times), the distance between the spacecraft pairs indicates that the current density determined from the gradient method (Figure 3b) will be underestimated accordingly. We therefore calculated the current density using the motion of each spacecraft separately as shown in Figure 3c. The negative peak of the first FAC current event is visible first in MMS4 and becomes stronger when it is observed by MMS3. It reaches maximum when it is observed by MMS2 and decays when it is observed by MMS1. These differences among the spacecraft suggest that the FAC event is a short time scale (a couple of seconds) event. Hence, these intense current sheet processes are transient and localized phenomena. They develop and decay in a couple of seconds, and the current layers have thicknesses of a couple of tens of kilometers.

During this event, the FPIs on MMS2 and MMS3 were operating in burst mode providing 3‐D distribution of electrons every 30 ms and ions every 150 ms. The −*V*_*b*_ component of the electron velocity moment from MMS3 (Figure 3d) and from MMS2 (Figure 3f) and the pitch angle sorted electron energy spectra from MMS3 (Figure 3e) and MMS2 (Figures 3g–3i) show that an enhancement in the low‐energy electrons of a few hundred eV streaming parallel to the ambient magnetic field coincides well with the interval of the FAC crossing as indicated by the vertical solid lines for the first FAC sheet crossing. (Note that due to limitations of the photoelectrons correction for this particular event, the electron moments demonstrate the qualitative signatures but are not useful for quantitative comparisons). The two‐dimensional cut of the electron distribution from MMS2 at 03:34:13.072 UT (Figure 3k) shows the electron beam parallel to the magnetic field, which suggests that these electrons are likely the carrier of the observed FAC. The low‐energy electron distribution then becomes more isotropic and decreases in energy (see Figure 3l). The second negative *J*_*B*_ peak is much weaker than the first but is still associated with a weak field‐aligned electron beam (see Figure 3m). Note that these beams and FAC activities happen in a closed‐field line region as can be seen by the existence of the hot plasma sheet electron and ion component near the high‐energy edge of the energy spectra. There is also some enhancement in the very low energy electrons simultaneous with the enhancement in the ion beam flux preceding the FAC event by about 0.6 s as indicated by the vertical dotted lines. MMS3 detected similar changes in the pitch angle distribution (not shown) except that MMS3 preceded MMS2 by about 0.45 s. Similar anisotropy in the field‐aligned components of low‐energy electrons, as described for this 03:34:12–03:34:14 UT FAC event, is observed also in the other FAC events shown in Figure 3 (vertical dotted lines).

## 5 Discussion

The PSBL is a dynamic region with field‐aligned ion beams or flows with a high‐speed component parallel to the ambient magnetic field. These beams are considered to be produced by acceleration due to reconnection or by acceleration in the distant tail current sheet [*Grigorenko et al.*, 2009, and references therein] as well as due to near‐tail reconnection and dipolarization fronts [*Zhou et al.*, 2012; *Birn et al.*, 2015]. The observed thinning of the plasma sheet and dipolarization taking place simultaneously suggest the formation of a near‐Earth X line in a closed‐field region for the first onset as suggested by *Sergeev et al.* [2008]. Both PSBL crossings (toward the lobe and toward the plasma sheet) showed a dominant disturbance in the *B*_*D*_ component, indicating a predominantly earthward FAC at the outer edge, and a tailward FAC at the inner region, as expected in a separatrix region. The dominant reversal of the FAC took place well inside the PSBL indicating that active reconnection remained in a closed‐field region during this interval.

The high‐resolution MMS measurement revealed for the first time the small‐scale fluctuating FACs and associated electron properties. Exclusively during the plasma sheet expansion (inward PSBL crossing), we observed highly structured low‐energy electron enhancements with energies less than 1 keV associated with spiky FACs on a time scale of a few seconds. They are observed mainly near the outer edge of the PSBL but in the presence of a hot plasma sheet electron component indicating closed‐field region phenomena. These spiky FAC/low‐energy electron enhancements are embedded within a fluctuating PSBL boundary of minute time scales. These fluctuating boundaries are also seen as enhancements in the energy of the cold ion beam indicating an enhancement of the drift velocity due to the enhanced dawn‐dusk electric field [*Sauvaud et al.*, 2004]. *Grigorenko et al.* [2010] reported that such minute‐scale fluctuations in the cold ion beam are created by the high‐energy ion beam‐induced flow shear that leads to the Kelvin‐Helmholtz (K‐H) instability and results in a large‐wavelength (5–20 *R*_*E*_) flux tube distortion.

Due to the planar geometry of the magnetic disturbance around 03:34:12 UT, we could identify the detailed spatial and temporal scales of short‐lived FACs that coincided with the tailward streaming electron beam. The timing analysis showed clearly that the FACs exist on an inward convecting flux tube with an enhanced electric field of about 4 mV/m. The FAC thickness was estimated to be 25 km. Due to the multicomponent plasma, i.e., a mixture of different composition and energy, and the effect of photoelectrons, and limited energy coverage of the plasma instrument, further quantitative comparisons between the field and the plasma moments are difficult for this event. Nonetheless, if we use the density of ions, i.e., 0.5/cm^3^ for H^+^ and 1.8/cm^3^ for O^+^, at 03:34 UT from HPCA to represent the average plasma properties, we can conclude that the thickness is well below the proton inertia length, 321 km, and is only about 3 times the electron inertia length of 8 km. Furthermore, there is a temporal evolution visible from the different profiles among the four spacecraft, suggesting that the entire event could have lasted only about 3 s. That is, the FAC signatures last only a couple of ion gyroperiods, estimated to be 1.0 s, based on the average magnetic field 65.3 nT. Hence, decoupling between ions and electrons are expected temporally as well as spatially for these current sheets, which explains the good consistency between the FACs calculated from the magnetic field and the electron beam signatures.

*Wright et al.* [2008] reported on an upward electron beams with a couple of 100 eVs in the PSBL at Cluster at 4–5 *R*_*E*_ that can be explained by low‐altitude (below 3200 km) acceleration. These beams are a more steady state structure, i.e., stable on a 1 min time scale and larger spatial scale (100 km at Cluster altitude), and correspond to much weaker current density, *j*∼6 nA/m^2^, if we map their values to MMS altitude assuming that *j*/*B* is conserved. Downward (earthward) electron beams accelerated by small‐scale (20–120 km), large‐amplitude (0.5–5 nT) kinetic Alfvén waves/spikes embedded in the overall 1 min fluctuations in the PSBL at 4–6 *R*_*E*_ were reported by *Wygant et al.* [2002]. We expect that such wave acceleration may create beams in both directions. One possible explanation of the observed electron beam might therefore be that such transient acceleration took place at an altitude between MMS and the ionosphere and that MMS observed the upward streaming part of the accelerated electron beams. A typical beam electron of 500 eV, corresponding to velocity of ∼13,000 km/s, will take more than 5 s to reach MMS from the ionosphere. This is comparable or longer than the time scale of the observed FAC events. Hence, it is more natural to conclude that acceleration processes took place well above the ionosphere. *Chaston et al.* [2012] reported that fast flows in the plasma sheet continually radiate kinetic Alfvén waves outward toward the lobe and the auroral oval. The observed spiky FAC and electron beam events are seen associated with the fluctuating low‐energy ion beam energy (Figure 2), as expected for enhanced convection electric fields. Although we cannot fully confirm it from the plasma instrument due to its energy coverage, this observation at least infers an existence of high‐speed ion flows. We suggest that such high‐speed ion flows or beams‐produced wave disturbance may have created the enhanced spiky electron beams to be detected at MMS.

## 6 Conclusions

Although MMS was in its commissioning phase, and hence, not all the instruments were operating on all four spacecraft, a number of instruments were taking data in burst mode and provided unprecedented detailed information on the multiscale properties of the near‐Earth PSBL during the substorms on 23 June 2015.

In particular, the detailed temporal and spatial scales of the small‐scale currents were identified for the first time with MMS's high temporal‐spatial measurements. Field‐aligned currents associated with the crossing of the separatrix region as well as spiky FAC just outside the separatrix region have been resolved.

The observed intense fine‐scale FACs are well below the ion scale and showed clear evidence that they are carried by low‐energy electrons most likely accelerated below the spacecraft, but well above the ionosphere, associated with the enhanced plasma jet or high‐energy ion beams in the plasma sheet boundary layer associated with an active near‐Earth reconnection region.

While some temporal and spatially localized ion beams at the PSBL have been resolved by Cluster, the fast electron beam properties in the PSBL have been for the first time resolved by MMS, which is essential for understanding multiscale properties of the effects of magnetotail reconnection.

## Supporting information



Supporting Information S1
