# Supplementary material for: Transient, small‐scale field‐aligned currents in the plasma sheet boundary layer during storm time substorms
Source: Geophys Res Lett. 2016 May 30;43(10):4841–9. doi: 10.1002/2016GL068768 (PMC5111425; doi:10.1002/2016GL068768)
Supplement: Supplementary file 1 — Supporting Information S1 [file GRL-43-4841-s001.pdf]

Geophysical Research Letters

Supporting Information for

Transient, small-scale field-aligned currents in the plasma sheet boundary layer during storm-time substorms

R. Nakamura,[1] V. A. Sergeev,[2] W. Baumjohann,[1] F. Plaschke,[1] W. Magnes,[1] D. Fischer,[1] A. Varsani,[1] D. Schmid,[1] T. K. M. Nakamura,[1] C. T. Russell,[3] R. J. Strangeway,[3] H. K. Leinweber,[3] G. Le,[4] K. R. Bromund,[4] C. J. Pollock,[4,5] B. J. Giles,[4] J. C. Dorelli,[4] D. J. Gershman,[4] W. Paterson,[4] L. A. Avanov,[4] S. A. Fuselier,[6,7] K. Genestreti,[6] J. L. Burch,[6] R. B. Torbert,[6,8] M. Chutter,[8] M. R. Argall,[8] B. J. Anderson,[9] P.-A. Lindqvist,[10] G.T. Marklund,[10] Y.V. Khotyaintsev,[11] B. Mauk,[9] I. Cohen,[9] D. N. Baker,[12] A. Jaynes,[1] R. E. Ergun,[12] H. J. Singer[13] J. A. Slavin,[14] L. Kepko,[4] T. E. Moore,[4] B. Lavraud,[15] V. Coffey,[16] Y. Saito[17]

[1]Space Research Institute, Austrian Academy of Sciences, Graz, Austria.
[2]St. Petersburg State University, St. Petersburg, Russia
[3]University of California, Los Angeles, California, USA
[4]Goddard Space Flight Center, NASA, College Park, Maryland, USA
[5]Denal Scientific, Healy, Alaska, USA.
[6]Southwest Research Institute, San Antonio, Texas, USA.
[7]University of Texas at San Antonio, San Antonio, Texas, USA.
[8]University of New Hampshire, Durham, New Hampshire, USA.
[9]Applied Physics Laboratory, Johns Hopkins University, Maryland, USA.
[10] Space and Plasma Physics, Royal Institute of Technology, Stockholm, Sweden.
[11] Swedish Institute of Space Physics, Kiruna, Sweden.
[12] University of Colorado, Boulder, Colorado, USA.
[13] NOAA Space Weather Prediction Center, Boulder, Colorado, USA.
[14] University of Michigan, Michigan, USA.
[15] Centre National de la Recherche Scientifique, Toulouse, France.
[16] NASA Marshall Space Flight Center, Huntsville, Alabama, USA.
[17] JAXA Institute for Space and Astronautical Science, Sagamihara, Japan.

Contents of this file

Figures S1 to S2
Tables S1 to S2

Additional Supporting Information (Files uploaded separately)

None

Introduction

The following figures contain supplementary information on the June 23, 2015 substorms from ground-based magnetograms (Figure S1) and from the MMS observations (Figure S2). The two supplementary tables are providing results from the minimum variance analysis of the magnetic field obtained by MMS (Table S1) and the orientation of the current sheet (Table S2) described in section 3 and 4 in the paper.

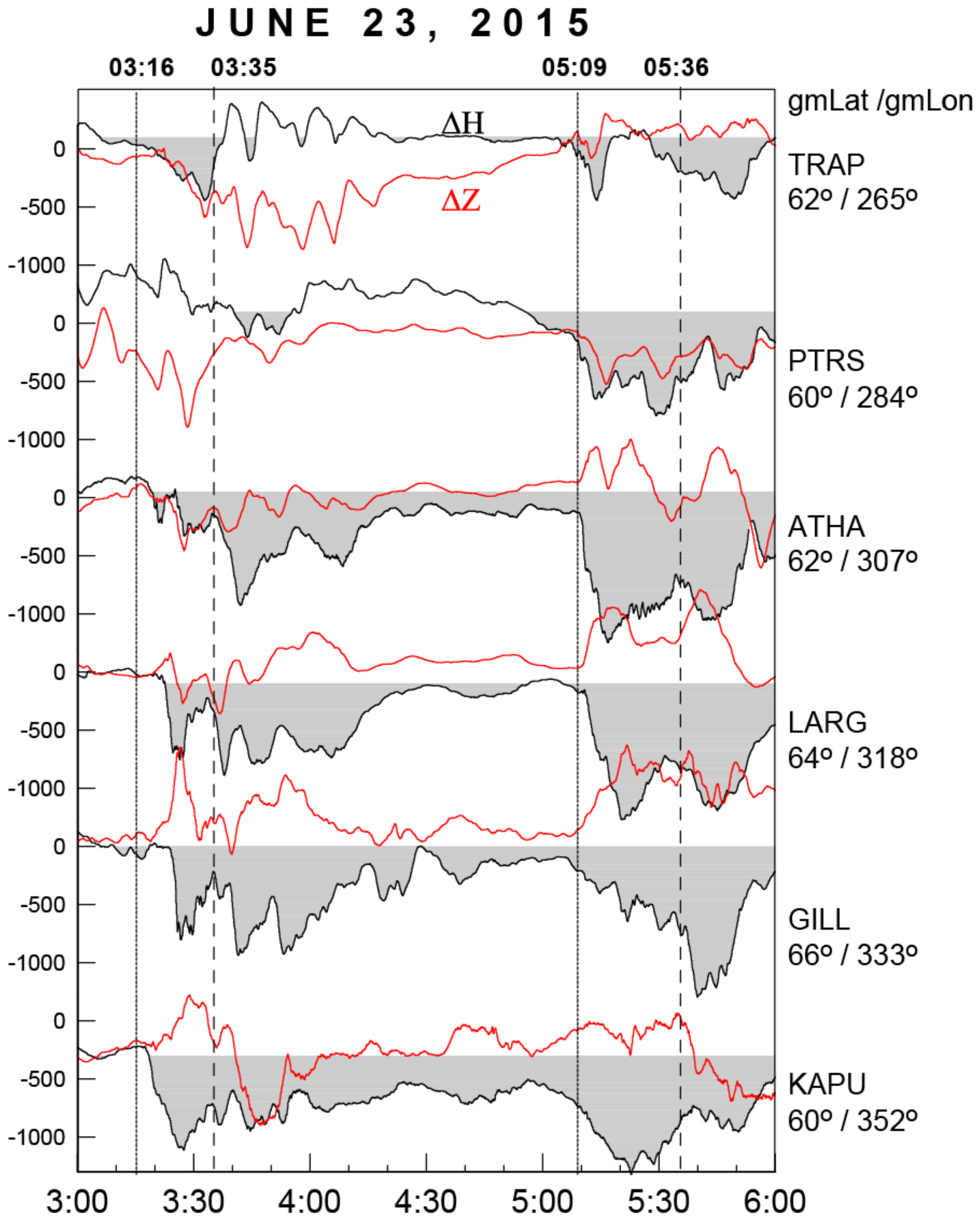

Figure S1. Geomagnetic disturbances in H component (black) and Z component (red) from 6 northern American stations: TRAP, PTRS, ATHA, LARG, GILL, and KAPU. Major enhancements in the westward electrojet with multiple intensification can be seen starting from 03:16 UT and 05:09 UT (solid vertical lines). The subsequent start times of major electrojet enhancements, 03:35 UT and 05:36 UT, are marked by dashed lines. For both substorms, MMS detected thinning of the plasma sheet in association with the first onset (solid lines) and expansion of the plasma sheet in association with the second onset (dashed lines), as explained more detail in the paper.

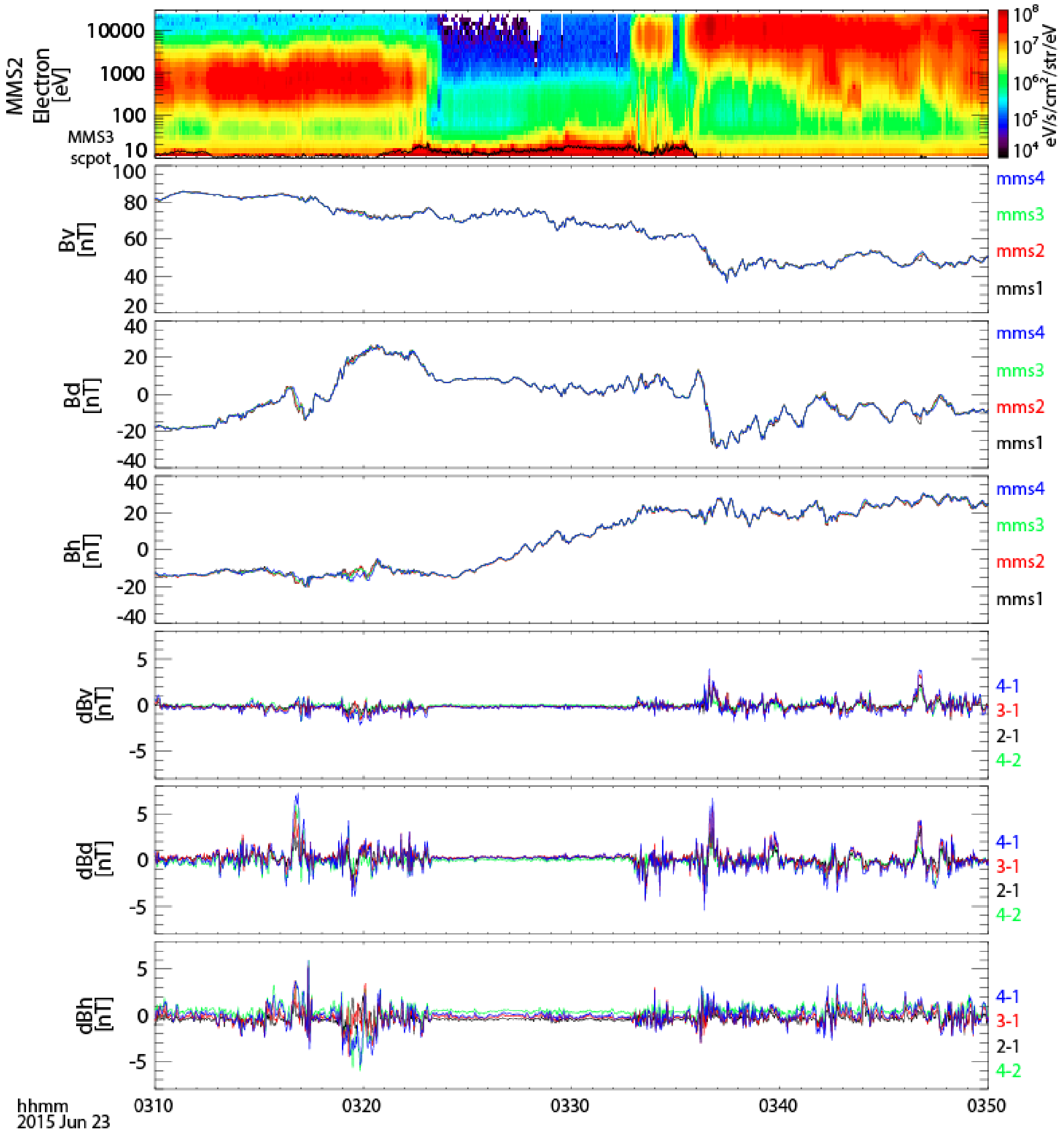

Figure S2. From top to bottom: Electron differential energy flux from MMS2 with spacecraft potential from MMS3 and magnetic field components: Bv, Bd, and Bz from the four MMS spacecraft and interspacecraft differences of these three components for different pairs of spacecraft (the number labeled correspond to the number of MMS spacecraft) during the plasma sheet thinning and expansion interval of the 03:16 UT substorm. The figure shows that maximum varying component during the time interval of the plasma sheet entry as well as exit is the Bd component. The magnetic field components compared between different two spacecraft pairs also differ largest for the Bd component, indicating that Bd varies both in space and time. Hence the magnetic field variations can be treated as near-1D current sheet.

SC	maxvar (U_1)	medvar (U_2)	minvar (U_3)	λ_1/λ_2	λ_2/λ_3	α^*
			2015-06-23/03:12:00 - 03:24:00			
MMS1	(0.20, 0.98, -0.05)	(0.79, -0.19, -0.58)	(0.57, -0.08, 0.82)	45.3	1.3	16.6
MMS2	(0.20, 0.98, -0.05)	(0.78, -0.19, -0.60)	(0.59, -0.08, 0.80)	46.3	1.4	16.9
MMS3	(0.19, 0.98, -0.04)	(0.81, -0.18, -0.56)	(0.55, -0.07, 0.83)	47.9	1.4	17.1
MMS4	(0.19, 0.98, -0.02)	(0.61, -0.14, -0.78)	(0.77, -0.14, 0.63)	51.1	1.4	17.1
			2015-06-23/03:33:00 - 03:37:00			
MMS1	(0.80, 0.60, 0.01)	(-0.59, 0.79, 0.18)	(0.10, -0.15, 0.98)	11.7	2.6	25.3
MMS2	(0.79, 0.61, 0.03)	(-0.60, 0.77, 0.19)	(0.09, -0.17, 0.98)	10.7	2.5	24.6
MMS3	(0.79, 0.61, 0.04)	(-0.61, 0.78, 0.14)	(0.05, -0.14, 0.99)	10.4	2.3	24.6
MMS4	(0.79, 0.61, 0.05)	(-0.61, 0.79, 0.06)	(-0.01, -0.08, 1.00)	10.3	2.3	24.7
			2015-06-23/03:34:13.5-15.5(MMS1), 12.5-14.5(MMS2,MMS3), 12.0-14.0(MMS4)			
MMS1	(0.49, 0.82, 0.29)	(-0.16, 0.41, -0.90)	(-0.86, 0.39, 0.33)	11.0	19.0	17.2
MMS2	(0.46, 0.82, 0.33)	(-0.35, 0.51, -0.79)	(-0.82, 0.25, 0.52)	31.7	23.3	19.1
MMS3	(0.39, 0.91, 0.13)	(0.04, 0.12, -0.99)	(-0.92, 0.39, 0.01)	53.2	10.8	8.8
MMS4	(0.39, 0.90, 0.20)	(-0.09, 0.25, -0.96)	(-0.92, 0.36, 0.18)	78.2	9.2	12.2

*angle between **-D** and U_1

Table S1. Result of minimum variance analysis for the plasma sheet thinning interval (Figure S2), plasma sheet expansion interval (Figures 2, S2), and during the small-scale current sheet interval (Figure 3). Spacecraft name, maximum variance direction, $\mathbf{U_1}$, intermediate variance direction, $\mathbf{U_2}$, minimum variance direction, $\mathbf{U_2}$, eigen value ratios, λ_1/λ_2 and λ_2/λ_3, and angle between **-D** and $\mathbf{U_1}$, where **D** is eastward direction, are shown in the table from left to right. The vectors are given in SM coordinate. The table shows that maximum variance direction for all three intervals for all four spacecraft is mostly aligned along east-west direction, as can be seen in the small angle of α.

	SM coordinate system	Explanation
$\mathbf{B_0}$	(-57.0, 23.5, 21.6)	1-minute average magnetic field between 03:34-03:35UT from MMS2
$\mathbf{b_0}$	(-0.87, 0.36, 0.33)	Unit vector of $\mathbf{B_0}$
**D**	(-0.47, -0.88, 0.00)	Eastward direction at 03:34 UT (MMS2 location)
**N**	(0.29, -0.16, 0.94)	$\mathbf{b_0} \times \mathbf{D}$
**B**	(-0.47, -0.88, 0.00)	$\mathbf{D} \times \mathbf{N}$

Table S2. Orientation of the 03:34 UT current sheet in the SM coordinate system. The normal to the field aligned current sheet, **N**, is tilted 19.4° from the north direction, **H.** The angle between $\mathbf{B}_0$ and **B** is 5.3°. Hence the *BDN* coordinate system can be regarded as a field-aligned current coordinate system, where *B* is the field-aligned direction and *D* is the field disturbance direction due to the field-aligned current sheet.
